# Antiparasitic Activities of Compounds Isolated from *Aspergillus fumigatus* Strain Discovered in Northcentral Nigeria

**DOI:** 10.3390/antibiotics12010109

**Published:** 2023-01-06

**Authors:** Oluwatofunmilayo A. Diyaolu, Gagan Preet, Adeshola A. Fagbemi, Frederick Annang, Guiomar Pérez-Moreno, Cristina Bosch-Navarrete, Olusoji O. Adebisi, Emmanuel T. Oluwabusola, Bruce F. Milne, Marcel Jaspars, Rainer Ebel

**Affiliations:** 1 Marine Biodiscovery Centre, Department of Chemistry, University of Aberdeen, Aberdeen AB24 3UE, UK; 2 Department of Pharmaceutical Chemistry, Faculty of Pharmacy, Lead City University, Ibadan 200005, Nigeria; 3 Fundación MEDINA, Parque Tecnológico de Ciencias de la Salud, Avenida del Conocimiento 34, Armilla, 18016 Granada, Spain; 4 Institut de Parasitiologia Biomedicina “Lopez-Neyra”, Consejo Superior de Investigaciones Cientificas (CSIC) Avda. Del Conocimiento 17, Armilla, 18016 Granada, Spain; 5 School of Biosciences, Aston University Birmingham, Birmingham B4 7ET, UK; 6 CFisUC, Department of Physics, University of Coimbra, Rua Larga, 3004-516 Coimbra, Portugal

**Keywords:** *Aspergillus fumigatus*, OSMAC, metabolomics, molecular docking, antitrypanosomal, antiplasmodial, in silico molecular docking

## Abstract

In this study, we explored a fungal strain UIAU-3F identified as *Aspergillus fumigatus* isolated from soil samples collected from the River Oyun in Kwara State, Nigeria. In order to explore its chemical diversity, the fungal strain UIAU-3F was cultured in three different fermentation media, which resulted in different chemical profiles, evidenced by LC-ESI-MS-based metabolomics and multivariate analysis. The methanolic extract afforded two known compounds, fumitremorgin C (**1**) and pseurotin D (**2**). The in vitro antiparasitic assays of **1** against *Trypanosoma cruzi* and *Plasmodium falciparum* showed moderate activity with IC_50_ values of 9.6 µM and 2.3 µM, respectively, while **2** displayed IC_50_ values > 50 µM. Molecular docking analysis was performed on major protein targets to better understand the potential mechanism of the antitrypanosomal and antiplasmodial activities of the two known compounds.

## 1. Introduction

Microbes continue to make a tremendous impact on natural product drug discovery. They produce many primary metabolites, such as amino acids, nucleotides, vitamins, and organic acids [1]. In addition, they produce secondary metabolites, which constitute a substantial part of the market’s pharmaceuticals today, to the extent that microbial natural products have become a principal source of drug lead compounds [2]. Various infectious diseases emerge worldwide, requiring a constant and broadened search for newer, more efficient bioactive molecules [2].

*Aspergillus* is a genus of all-pervasive fungi that are beneficially and morbidly important. The members of the genus *Aspergillus* are urbane and ubiquitous constituents of different habitats [3]; this is because they can colonise a broad range of substrates. A wide range of secondary metabolites has been isolated from this genus [4]. These metabolites have been linked to 24 biosynthetic families. Examples include alkaloids, benzoquinones, flavonoids, phenols, anthraquinones, steroids, terpenoids, tetralones, and xanthones [5]. Some bioactivities of compounds isolated from *Aspergillus fumigatus* have been studied and reported in the literature, including antibacterial [6], antinematodal [7,8], antiprotozoal [9,10], antifungal [11], antiviral [12], anticholinesterase [13], and cytotoxic activities [14].

Parasitic diseases concern health and human life, particularly in the developing world. On a global scale, parasitic diseases are responsible for almost one million deaths yearly [15]. They remain the significant killers of children in low-income nations [16,17,18]. In 2019, the World Health Organisation (WHO) tagged malaria as a significant parasitic disease, causing 409,000 deaths, most of whom were African children below the age of five [19]. However, it is thought that many cases are undiagnosed, and therefore unpublished, so the actual figure may well be higher. In tropical regions, the combination of warm and humid climates with exponential population growth and poverty contributes to the rise in disseminating parasitic infections [20], usually mediated by vectors such as mosquitoes and ticks. Protozoans belonging to the *Plasmodium* group are the causative agents for malaria and cause worldwide deaths [16,17]. Antiparasitic drugs currently in use suffer from several drawbacks, as they tend to be expensive, their side effects may be severe, they may be challenging to administer, or drug resistance towards them may be rapidly expanding [21,22,23].

For instance, African Trypanosomiasis, one of the neglected tropical diseases caused by *Trypanosoma brucei,* is transmitted by the tsetse fly (*Glossina* species) found exclusively in sub-Saharan Africa [24]. The parasite, which comprises two physically identical subspecies, affects people in two different ways. While the *T. rhodesiense* strain produces a more severe acute African trypanosomiasis in eastern and southern Africa, the *T. gambiense* strain causes a slowly progressive disease in western and central Africa [25]. An estimated more than 60 million people in sub-Saharan Africa have been affected [7].

However, the ability of *Aspergillus fumigatus* to biosynthesise bioactive natural products could result in previously unexplored therapeutic options to fight these parasites. Consequently, we decided to extend our investigation of *Aspergillus fumigatus* by screening the isolated compounds for their potential antimalarial and antitrypanosomal activities using in silico molecular docking and in vitro studies. Furthermore, we studied the effect of altering the growth conditions of the fungus on their metabolic profiles using LC-ESI-MS-based metabolomics followed by multivariate analysis. Herein, this work presents for the first time the antiplasmodial and trypanocidal activities of fumitremorgin C (**1**) and pseurotin D (**2**). To identify the potential connections between the binding model and the antiparasitic properties, we employed molecular docking to fit compounds **1** and **2** into the active site of the target enzyme.

## 2. Results and Discussion

### 2.1. Characterisation and Identification of Strain UIAU-3F

**Figure 1:**
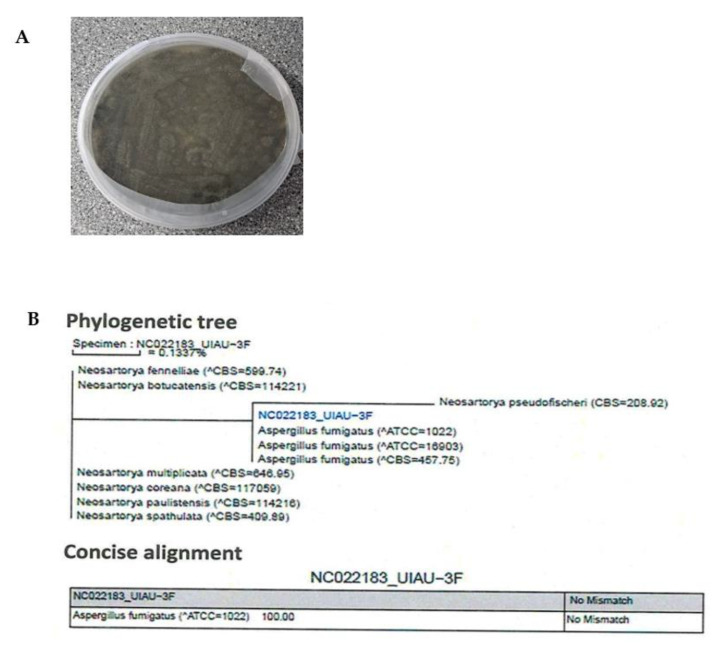
(**A**) Colony characteristics of *Aspergillus fumigatus* UIAU-3F grown on ISP2 agar at 28 °C for 7 days. (**B**) Phylogenetic tree of *Aspergillus fumigatus* UIAU-3F.

The identification of UIAU-3F was conducted based on the morphology and phylogenetic analysis. Incubation was performed at 28 °C for 10 days, upon which strain UIAU-3F formed colonies on ISP2 agar plates displaying characteristic hyphal structures (Figure 1A). The ITS gene region of the ribosomal DNA of the strain was amplified by PCR- and sequenced. The phylogenetic tree (Figure 1B) constructed from the ITS gene sequence indicated that UIAU-3F belonged to the genus of *Aspergillus* with the highest similarity to *A. fumigatus* (100%, accession number: EF66998).

### 2.2. Non-Targeted Multivariate Analysis

**Figure 2:**
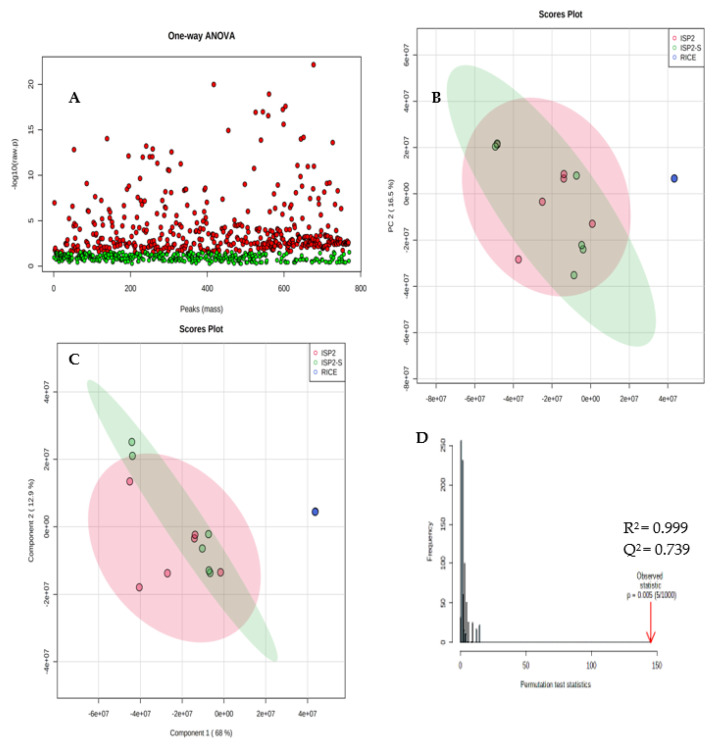
(**A**) Multivariate analysis. ANOVA results show statistically significant metabolites in red. (**B**) Principal Component Analysis (PCA) score plot. Green circles correspond to ISP2−S extract; pink circles correspond to ISP2 extract; blue circles correspond to RICE extract. (**C**) Partial least squares discriminant analysis PLS−DA model. Colour coding as before. (**D**) A permutation test plot.

After fungal identification and culturing of the strain in the different growth media, the extracts obtained were subjected to comparative metabolomics. Supervised statistical PCA, PLS-DA, permutation tests, and heat maps were used to compare the presence or absence and relative abundance of parent ions in each extract. Using metaboAnalyst software, the peak lists generated from filtered positive and negative mode mass spectral data were analysed. Ions present in solvent and media blanks were eliminated from the analysis to avoid uninformative skewing of the results. We detected 770 features between all three extracts, with each feature representing a distinctive combination of the *m/z* value and chromatogram peak characteristics. ANOVA was used to compare and determine if there was a statistically significant difference between the three fungal extracts (ISP2, ISP2-S, and RICE). The *p*-value threshold was set to 0.05 (default). Metabolites with a *p*-value below the threshold are shown in red and are deemed statistically significant. Of the metabolites predominantly originating from the RICE extract, 433 are statistically significant and 337 are insignificant (Figure 2A).

The PCA score plot enables the visualisation of how the three extracts are related to one another. Extracts are clustered according to their similarity, with the more distinct sample groups showing more separation. The score plot comprising six replicates of the RICE, ISP2, and ISP2-S extracts in positive and negative mode ionisations was acquired, as shown in Figure 2B. Metabolites obtained from the RICE media (RICE 1–6) were separated from metabolites obtained from ISP2 (1–6) and ISP2-S medium by PC1. However, metabolites from ISP2 positively correlate with ISP2-S; this may be because of the close similarity in media culturing conditions and therefore suggests similarities in their secondary metabolites. The first two components, PC1 and PC2, explained 68.2% and 16.5% of the total variance, respectively.

PLS−DA sharpens the separation between the extracts as it can perform both classification and metabolite selection. To identify the metabolites that contribute to the different metabolomic patterns, the PLS−DA model was applied (Figure 2C). As PLS−DA analysis is prone to overfitting, it is essential to validate PLS−DA analysis by conducting cross-validation or a permutation test. Cross-validation of Q^2^ and R^2^ is used to select the optimal number of compounds used in the PLS−DA model for classification. This model obtained an R^2^ of 0.993 and a Q^2^ of 0.739. The R^2^ suggests that the model can be considered to have a substantial predictive ability and the Q^2^ obtained is close to the R^2^, indicating that the data fit the model (Figure 2D).

**Figure 3:**
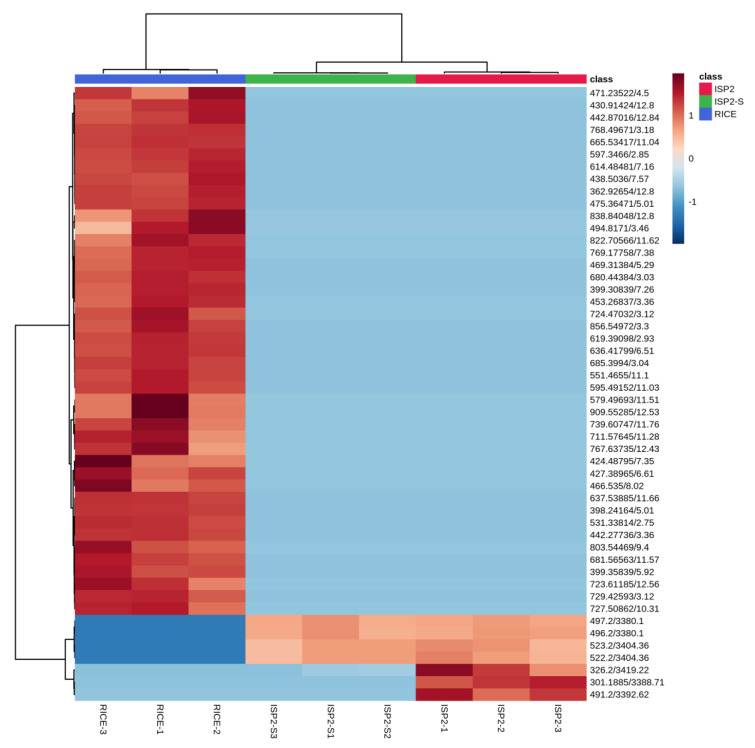
Hierarchical clustering of detected ions in ISP2 extract (red), ISP2-S extract (green), and RICE extract (blue). Heatmap displays relative abundance (low, blue; high, red) of respective features.

A heatmap was generated by visualising the intensities of the metabolites in order to determine the abundance of the secondary metabolomes in the three different extracts (Figure 3). The samples were conducted in triplicate. Multiple red bands indicate a rich secondary metabolome with a high diversity of metabolites, while deep blue bands exemplify a more limited set of secondary metabolites. The RICE culture extract showed the richest metabolome of the three extracts, as is evident from the heatmap display (Figure 3).

#### Metabolites Identification

**Figure 4:**
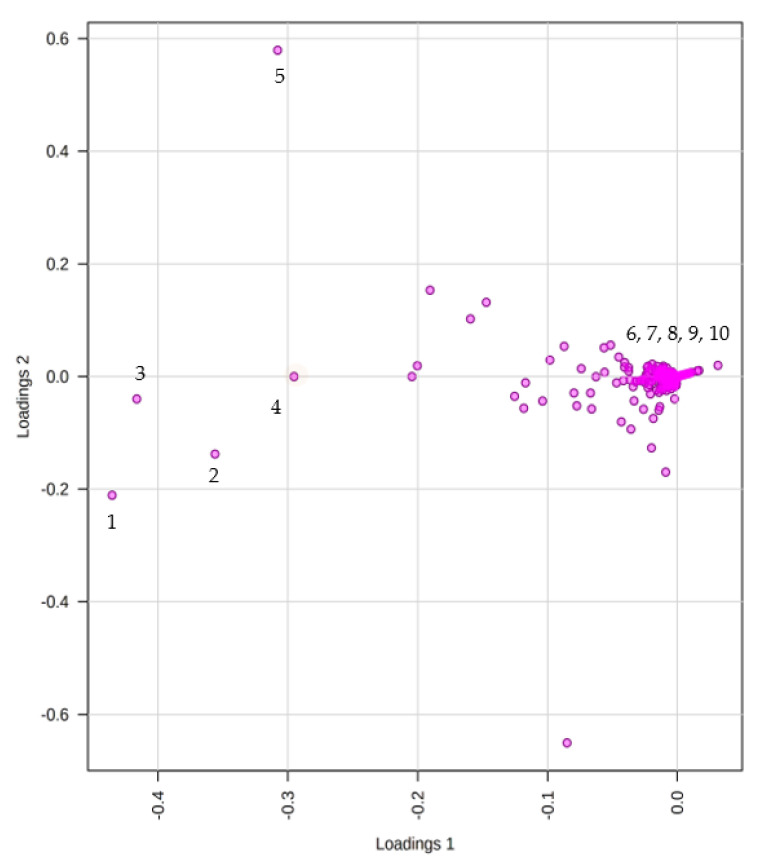
Principal Component Analysis (PCA) loadings score plot to display discriminant meta-bolites.

The loadings plot generated (Figure 4) enables the identification of the metabolites within the three extracts responsible for the driving patterns seen in the score plot. Metabolites displaying similar information are grouped, indicating a correlation between the ISP2 and ISP2-S extracts. Compounds labelled **1**–**5** in the loading plot can be found in both ISP2 and ISP2-S extracts. An inverse correlation is displayed between the RICE and ISP2 extracts, as metabolites of the RICE extract are positioned on the opposite side of the plot of origin and farther away (compounds **6**–**10**).

Potential biomarkers were initially identified by their molecular weights and fragmentation patterns using mass spectrometry. The precise mass of each differential metabolite was searched using different databases (Reaxys, NPAtlas, PubChem) to confirm its identity, possible molecular formula, and chemical composition (Table 1).

### 2.3. Structural Elucidation of Isolated Compounds

The fungal UIAU-3F extract obtained from the RICE fermentation medium was analysed using HR-LCMS and ^1^H NMR spectroscopy. This extract was selected for fractionation and subsequent purification based on the diversity of metabolites dereplicated in the crude extract (Table 1) and its larger production mass compared to the ISP2 and ISP2-S extracts (Figure 4). The ^1^H NMR spectrum showed a wide range of interesting signals that indicated the chemical diversity of this extract. The UIAU-3F extract was fractionated and purified, as described in Section 3. Two known compounds, fumitremorgin C (**1**) [33] and pseurotin D (**2**) [34], were isolated and identified by comparing their experimental NMR and HRESIMS data (Figures S2–S6 and S8–S12) with the published literatures.

### 2.4. Determination of Antitrypanosomal and Antiplasmodium Activity Using Molecular Docking

In order to ascertain the antitrypanosomal and antiplasmodial activities of compounds **1** and **2**, molecular docking studies were conducted. Two enzymes were used in the docking study: Cruzain, the principal papain-like cysteine protease of *Trypanosoma cruzi*, and l-lactate dehydrogenase from *Plasmodium falciparum*. Cruzain is crucial for the survival and the multiplication of the parasite *Trypanosoma cruzi* [33], while l-lactate dehydrogenase is considered a potential molecular target for antimalarials due to the parasite’s dependence on glycolysis for energy production [34]. To gain an insight into the differences in binding between the compounds and these proteins, rigid receptor docking was performed.

Docking poses were analysed and compared to benznidazole and chloroquine standards for antitrypanosomal and antiplasmodial activities, respectively. The first docking was performed on the crystal structure of cruzain from *Trypanosoma cruzi* (PDB: 3I06) [35]. In contrast, the second docking was performed on the crystal structure of L-lactate dehydrogenase from *Plasmodium falciparum* (PDB: 1LDG).

**Figure 5:**
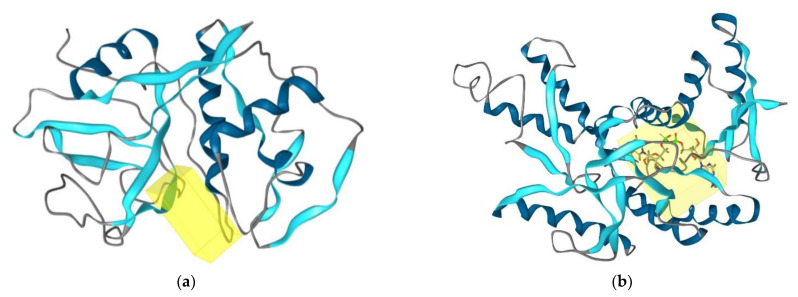
(**a**) Binding site (yellow colour) of cruzain from *Trypanosoma cruzi.* (**b**) Binding site (yellow colour) l-lactate dehydrogenase from *Plasmodium falciparum*.

Compounds **1** and **2** were subjected to docking analysis, and the specificities of their interaction with these targets were identified, as shown in Figure 5 Based on binding energies and interacting residues, the best-docked complexes were obtained. Docking poses were analysed and compared to the standards. In both the two molecular docking studies, **1** and **2** docked very well compared to the standards (Table 2 and Figure 5).

**Figure 6:**
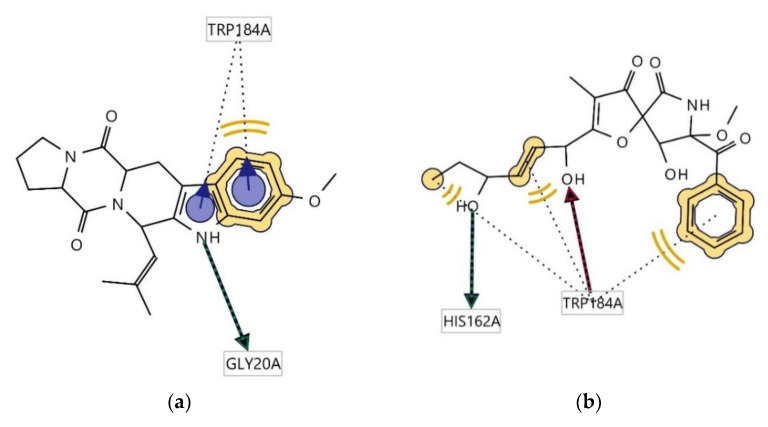
Ligplots showing interacting residues of cruzain complex with compounds **1** (**a**) and **2** (**b**). Red arrow dotted lines, HBAs; green arrow dotted lines, HBDs; Yellow lines, H; Purple arrows, AR interactions.

Ligplots in Figure 6 show significant amino acid residue interactions with the two ligands. In both cases, Trp184(A) was found to be involved in hydrophobic interactions. Compound **1** interacts with cruzain, thus acting as a hydrogen bond donator (HBD) at the receptor site interacting region involving residue Gly20A. Cruzain’s residue Trp184(A) was also involved in aromatic interaction with the indole moiety of the structure. The interaction of **2** with cruzain involves both hydrogen bond acceptor (HBA) interaction with Trp184A and HBD with His162A.

**Figure 7:**
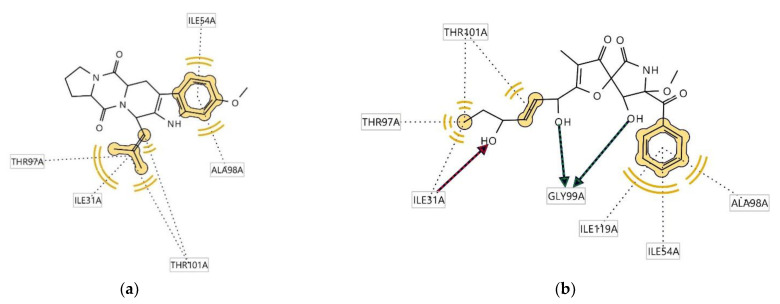
Ligplots showing interacting residues of l-lactate dehydrogenase complex with compounds **1** (**a**) and **2** (**b**). Red arrow dotted lines, HBAs; green arrow dotted lines, HBDs; yellow lines, H interactions.

Ligplots in Figure 7 show significant amino acid residue interactions with the two ligands. In both cases, Thr97(A), Ile31(A), Thr101(A), Ala98(A), and Ile54(A) were found to be involved in hydrophobic interactions. The interaction of compound **2** with l-lactate dehydrogenase involves both HBA interaction with Ile31(A) and HBD with Gly99(A).

### 2.5. In Vitro Antiparasitic Activity

**Figure 8:**
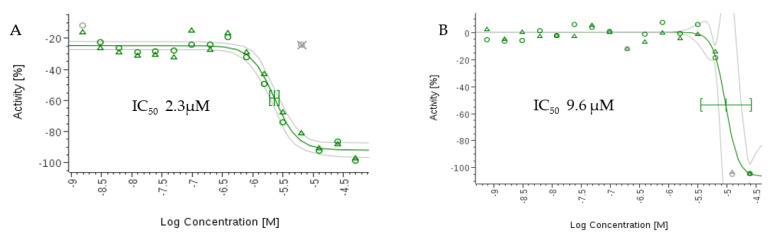
(**A**) Dose−dependent IC_50_ curve of **1** for *P. falciparum* 3D7. (**B**) Dose−dependent IC_50_ curve of **1** for *T. cruzi* Tulahuen.

The compounds were tested using the in vitro β-Β-galactosidase-transgenic *T. cruzi* and the *P. falciparum* 3D7 lactase dehydrogenase assay. Although compound **2** showed activity in the in silico studies, it displayed only moderate antitrypanosomal and antiplasmodial activities in vitro with IC_50_ values greater than 50 µM compared to the standards (Table 3). This may be due to several factors, such as low cell permeability to the parasite cells, interstitial hypertension, and metabolic degradation. However, **1** displayed significantly higher activities than **2** against *T. cruzi* and *P. falciparum*, with IC_50_ values of 9.6 µM and 2.3 µM ( Figure 8). The IC_50_ values are higher than the standard: 2.6 μM for benznidazole and 0.017 µM for chloroquine.

Compound **1** showed moderate antiparasitic activity against *T. cruzi* Tulahuen C4 and *P. falciparum*; to our knowledge, this is the first account of the inhibitory activity of this compound against these two tropical parasites. Watts et al. suggest that the peroxide ring present in the fused spiro-pentacyclic diketopiperazines is not the bioactive pharmacophore. They screened the diketopiperazines verruculogen TR-2, fumitremorgin B, 12,13-dihydroxyfumitremorgin C, and cyclotryprostatin A isolated from *Aspergillus fumigatus* for their trypanocidal potential by testing for inhibitory activity against a panel of cysteine proteases. Fumitremorgin B and 12,13-dihydroxyfumitremorgin C exhibited IC_50_ values of 0.2 μM and 7.4 μM, respectively. The abundant enzyme isoform in the parasite is suggested to be rhodesain (also referred to as trypanopain or brucipain) [21], although it is believed that members of the rhodesain family of enzymes are not crucial for the sustenance of the parasite. However, they play essential roles in the second stage of host infection, parasite migration across the blood–brain barrier [36]. We have evaluated **1** and **2** against the major protein targets to determine whether the compounds have an affinity for the essential receptors connected to antiparasitic mechanisms. Molecular docking analysis indicated that these compounds gave potential binding consistent with antitrypanosomal and antiplasmodial activities.

## 3. Material and Methods

### 3.1. General Procedure

Bruker Avance III 400 and 600 MHz (equipped with a liquid nitrogen-cooled Prodigy cryoprobe) spectrometers were used to record NMR spectra at the University of Aberdeen, UK. Spectra were acquired at 25 °C and chemical shifts were referenced to the residual solvent peaks: 3.31 and 49.1 ppm (CD_3_OD), 2.50 and 39.52 ppm (DMSO-*d*_6_), and 4.78 ppm (D_2_O). An edited Heteronuclear Single Quantum Coherence (HSQC) experiment was used to determine the multiplicities of the peaks in the ^13^C NMR spectrum. Deuterated solvents were acquired from Cambridge Isotope Laboratories, Inc., UK. High-resolution electrospray mass spectra were obtained using a Bruker maXis II electrospray ionisation quadrupole time-of-flight (ESI-qToF) mass spectrometer coupled with an Agilent reversed-phase HPLC system. Solvents for HPLC, i.e., acetonitrile and methanol, were obtained from Rathburn Chemicals Ltd., Scotland, UK, while water was obtained from a Milli Q water system.

### 3.2. Aspergillus fumigatus Collection

**Figure 9:**
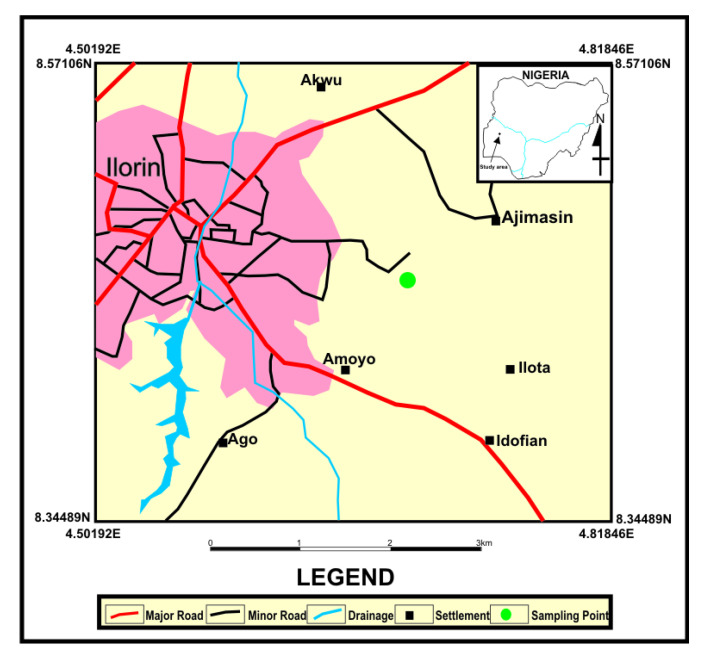
Map showing sample collection site.

Soil samples were collected from the River Oyun in Kwara State, Nigeria. The fungal strain, UIAU-3F, was isolated from the soil sediment. The collection site, with coordinates (8°27′57.8412″ N 4°40′3.4428″ E) (Figure 9), in Ilorin Kwara State, Nigeria, was highly contaminated with heavy metals [37,38]. The strains were isolated by Dr Olusoji Olusegun Adebisi from the University of Ilorin, Kwara State, and stored at the university’s microbiology department with the voucher specimen number UIL-0022.

### 3.3. Chemical Reagents

The high analytical grade chemicals and other reagents used in this study were all acquired from Fisher Scientific UK Ltd. in Leicestershire, UK.

### 3.4. DNA Isolation and Molecular Identification

The pure strain was preserved in duplicates in 25% aqueous glycerol stock and stored at −80 °C at the Marine Biodiscovery Centre, University of Aberdeen. Subsequently, it was inoculated in 100 mL of ISP2 liquid medium for 5 days; the pellet was collected in a 2 mL microcentrifuge tube and the supernatant was discarded. The DNA was extracted using the QIAGEN-DNeasy UltraClean Microbial Kit following the manufacturer’s quick start protocol. Whole-genome sequencing of strain UIAU-3F was conducted by the National Collection of Industrial, Food and Marine Bacteria (NCIMB), Bucksburn, Aberdeen.

For assembly, the information collected from the single SMRT sequencing cell was used. To create the final genomic sequence, the raw reads were improved upon and assembled using the hierarchical genome assembly method (HGAP) [39].

### 3.5. Fermentation and Extraction

The isolated *Aspergillus fumigatus* strain was fermented under three different culturing conditions: solid rice medium (RICE), liquid ISP2 (malt extract 10 g L^−1^, yeast extract 4 g L^−1^, and glucose 4 g L^−1^) medium with continuous shaking (ISP2-S), and static liquid ISP2 medium. The solid medium fermentation (RICE medium) was performed using 100 g of commercial rice and 110 mL water in a 1 L Erlenmeyer conical flask autoclaved for 20 min at 121 °C. After cooling, 2 mL of the fungal spore suspension was inoculated into the flask and incubated at 28 °C under static conditions for 30 days. Liquid fermentations were performed in 2 L Erlenmeyer flasks containing 1.5 L ISP2 medium. After inoculation, they were allowed to grow for 10 days at 30 °C while shaking at 150 rpm. The mycelium was removed from the liquid culture by filtration and soaked in 100% methanol for 24 h, while the liquid fungal medium was treated with the HP 20 resin (30 g L^−1^). The combined methanol extract from the mycelium and the liquid fungal medium was evaporated until dryness under reduced pressure, before 500 mL of EtOAc was added to the solid rice media, and EtOAc extract was filtered and dried with a vacuum rotary evaporator (BÜCHI R-114, Switzerland) at 40 °C.

### 3.6. Metabolic Profiling and Multivariate Statistical Analysis

A final concentration of 0.1 mg mL^−1^ of crude extracts was prepared using 100% methanol, centrifuged and injected into a Phenomenex Kinetex XB-C18 (2.6 µm, 100 × 2.1 mm) column. Samples were analysed using a Bruker maXis II electrospray ionisation quadrupole time-of-flight (ESI-qToF) mass spectrometer coupled with an Agilent 1290 UHPLC system. The elution conditions were 5% MeCN + 0.1% FA to 100% MeCN + 0.1% FA in 15 min. The Bruker maXis II had a mass range of 100–2000 *m*/*z* and was equipped with the positive and negative mode conditions. The internal settings included 4.5 kV capillary voltage, 4.5 bar nebuliser gas, 12.0 L min^−1^ dry gas, and a dry temperature of 250 °C. MS/MS fragmentation experiments were adjusted under Auto MS/MS scan mode with no stepwise collision. A mass of *m*/*z* 248.96 was used for the negative mode and an *m*/*z* of 226 for positive mode calibration. The external reference lock mass (sodium formate) was infused at a constant flow of 0.18 mL h^−1^.

Using MSConvert software, the HPLC-MS/MS data acquired from the crude extracts were converted from data analysis (.d) to.mzXML file format. The mzXML files were further processed using MZmine software [40]. The following parameter modules were used: mass detection (RT 2.5–35 min, centroid); chromatogram builder (MS level 1; minimum height 1.0 × 104; minimum time 0.5 min; *m*/*z* tolerance 10 ppm); deconvolution of the spectra (Savitzky–Golay algorithm); isotopic peaks grouper (*m*/*z* tolerance 10 ppm; retention time (RT) tolerance 0.1 min); duplicate peak filtering; smoothing; data alignment (join aligner; *m*/*z* tolerance 10 ppm; RT tolerance 0.5 min); gap-fling (intensity tolerance 20%; *m*/*z* tolerance 10 ppm; RT tolerance 0.5 min); and peak filtering range (0.00–0.5 min). After processing the data, peaks in several samples with the same RT and *m*/*z* were assumed to come from the same component. Furthermore, MetaboAnalyst 4.0 [41] was used for multivariate statistical analysis. The resulting peak intensity table was uploaded to MetaboAnalyst and, during the data integrity check, any missing values were replaced with small values. Prior to being auto-scaled and normalised to the median, the data were log-transformed. Heat maps of chemical profiles were constructed using hierarchical clustering and Principal Component Analysis (PCA).

### 3.7. Isolation of Metabolites

The ISP2-S crude extract (1.15 g) was sequentially fractionated using a modified Kupchan liquid–liquid partitioning method [42]. The crude extract was partitioned between an aqueous (water fraction; 0.30 g) and organic (dichloromethane fraction; 0.62 g) solvent layer; this afforded two fractions (A and B). Fraction B was then purified by solid-phase extraction (SPE) with the aid of a 10 g pre-packed Phenomenex strata C18-E (55 μm, 70 Å) column as the stationary phase. Aqueous methanol (25%, 50%, 100%, and 100% + 0.1% TFA) was the mobile phase. The fraction FD-100% MeOH (32.3 mg) was purified by HPLC and a Phenomenex C18 Luna phenyl-hexyl (250 × 10 mm 10 μ micron) column. The mobile phase was made of methanol, water, and trifluoroacetic acid (TFA) (0.05%). A linear gradient solvent from 60:40 H_2_O/MeOH to 0:100 MeOH ran for 30 min with a flow rate of 1.8 mL min^−1^ to afford compounds **1** (4.42 mg, *t_R_* = 13.9 min) and **2** (3.80 mg, *t_R_* = 15.6 min). Optical rotations of both compounds were recorded using a PerkinElmer model 241 polarimeter and compared to that obtained from the literature.

### 3.8. Molecular Docking

The crystallographic protein structures used for docking were PDB: 3I06 [43], which is cruzain from *Trypanosoma cruzi*, and PDB: 1LDG [44] of l-lactate dehydrogenase from *Plasmodium falciparum*. These were obtained from the Protein Data Bank and used to perform docking simulations. The receptor sites were predicted using LigandScout (Inte: Ligand) Advanced software [35], which identifies putative binding pockets by creating a grid surface and calculating the buriedness value of each grid point on the surface. The resulting pocket grid consists of several clusters of grid points, rendered using an iso surface connecting the grid points. The iso surface represents an empty space suitable for creating a pocket.

Molecular docking analysis was performed using Autodock Vina v.1.2.0 (The Scripps Research Institute, La Jolla, CA, USA) docking software [45]. All ligands and protein structures were prepared using the Dock Prep tool with default parameters in Chimera 1.16 [46]. The net charges were set to neutral for all ligands. The box centre and size coordinates in Angstrom units for PDB: 3I06 were −7.1 × −33.4 × −0.6 and 11.2 × 13.3 × 16.7 and for PDB: 1LDG, 32.9 × 25.7 × 34.6 and 20.4 × 17.8 × 22.0 around the active site. The search parameters used for production runs were: number of binding modes = 10, exhaustiveness = 32, and maximum energy difference = 3 kcal mol^−1^. The results were tested for convergence at exhaustiveness 8, 16, 24, and 32 keeping all the parameters fixed. LigandScout (Inte: Ligand) Advanced software [35] was used to visualise and calculate protein–ligand interactions.

### 3.9. In Vitro Antitrypanosomal Activity

The procedure used for the in vitro -β-Galactosidase transgenic *T. cruzi* experiment was previously described by Annang and colleagues [47]. It entails *T. cruzi* Tulahuen C4 strain expression of the galactosidase gene (LacZ), with L6 rat skeletal muscle cells serving as the host cells. At 37 °C and 5% CO_2_, they were grown in RPMI-1640 that had been supplemented with 10% iFBs, 2 mM l-glutamine, 100 U mL^−1^ penicillin, and 100 g mL^−1^ streptomycin. To test the compounds, *T. cruzi* amastigote-infected L6 cell culture (2103 infected L6 cells per well) was dispersed into 384-well assay plates (1, 2). The initial concentration was 25 M, with each molecule examined in triplicate in 16-point dose–response curves (1/2 serial dilution). The plates were incubated for 96 h at 37 °C. Following the addition, 1.5 L of 100 M CRPG and 0.1% NP40 were further incubated at 37 °C for 4 h in the dark. The absorbance at 585 nm was measured using an EnVision plate reader (PerkinElmer, Waltham, MA, USA). The in-plate was used to normalise the test. Benznidazole at 10 μg mL^−1^ was used as the negative control and 0.167% DMSO as the positive control.

### 3.10. Plasmodium falciparum 3D7 Lactase Dehydrogenase In-Vitro Assay

The *P. falciparum* 3D7 strain parasites were cultured in a freshly collected type O positive (O+) human erythrocyte. The assay was executed using a previously described standardised method by Annang et al. [48]. After 72 h of incubating the parasites with the test compounds (**1**, **2**), the synthetic cofactor APAD+, which is specific to the parasite LDH enzyme [49], was used to quantify the proportions of parasite viability by measuring the activity of this intracellular enzyme that is released upon lysis of the parasites. The assay was performed in triplicate using a 16 points dose–response curve (1/2 serial dilution) with starting concentrations of 50 μM in 384-well plates. Each plate had 100 nM of chloroquine as a negative control and a parasite culture media as a positive growth control. LDH activity was assessed after the plates had been incubated for 72 h, frozen for 4 h, and then thawed at room temperature for 1 h. To perform this, 70 mL of a freshly made solution containing 100 mM Tris-HCL at a pH of 8.0, 143 mM sodium l-lactate, 143 mM APAD, 178.75 mM NBT, 1 g mL^−1^ diaphorase, and 0.7% Tween 20 was poured into the plates. After a 10 min incubation period at room temperature and a gentle shake of the plates to ensure uniformity, the absorbance was measured at 650 nm. The absorbance in this test was measured using the EnVision plate reader (PerkinElmer, Waltham, MA, USA).

### 3.11. Statistical Analysis

All experiments were performed in triplicate and results were expressed as the mean ± SEM. The statistical significance of the means differences was confirmed by analysis of variance (ANOVA) with Duncan’s post hoc tests, with *p*-values < 0.05 considered statistically significant.

## 4. Conclusions

We investigated the metabolomic diversity and the antiparasitic activity of fumitremorgin C (**1**) and pseurotin D (**2**) isolated from *Aspergillus fumigatus* found in soil samples collected from the River Oyun in Kwara State, Nigeria. Fermentation of the fungus under different growth conditions (OSMAC approach) stimulated their metabolite production, as evidenced by the LC-HRMS metabolomic profiling. In vitro studies revealed **1** as the most active against *Trypanosoma cruzi* and *Plasmodium falciparum,* with IC_50_ values of 9.6 and 2.3 µM, respectively. Molecular docking supported this observation by predicting binding modes and affinities in line with the experimental activity profile. As a result, we feel this compound warrants further investigation, including in vivo and mechanism of action studies.

## Figures and Tables

**Table 1:** Significant differential metabolites identified (*p*-value < 0.05 VIP).

Metabolite	[M+H]^+^	t_R_ (min)	MF	Compound	Source	Ref
1	480.1595	4.5	C_27_H_33_N_3_O_5_	Fumitremorgen B	ISP2, ISP2-S	[26]
2	496.2338	3.8	C_27_H_33_N_3_O_6_	Spirotryprostatin C	ISP2, ISP2-S	[27]
3	528.5353	7.7	C_27_H_33_N_3_O_8_	Spirotryprostatin E	ISP2, ISP2-S	[28]
4	524.3704	5.6	C_33_H_49_NO_4_	Anthcolorin E	ISP2, ISP2-S	[29]
5	522.3404	3.3	C_33_H_47_NO_4_	Anthcolorin H	ISP2, ISP2-S	[29]
6	478.5882	7.1	C_28_H_34_N_2_O_5_	Fumitremorgen B derivative	RICE	[30]
7	431.4423	6.9	C_22_H_25_NO_8_	Pseurotin D	RICE	[31]
8	381.4316	6.2	C_21_H_23_N_3_O_4_	Cyclotryprostatin C	RICE	[28]
9	379.4151	6.4	C_21_H_21_N_3_O_4_	Cyclotryprostatin D	RICE	[32]
10	511.6752	4.5	C_27_H_33_N_3_O_7_	Verruculogen	RICE	[32]

t_R_—retention time; MF—molecular formula; Ref—reference.

**Table 2:** Docking analysis of two ligands on two different protein receptors with respect to benznidazole and chloroquine standard.

Compounds	Docking Score (-) (kcal mol^−1^)	Docking Score (-) (kcal mol^−1^)
	(PDB: 3I06)	(PDB: 1LDG)
	Cruzain from *Trypanosoma cruzi*	l-lactate Dehydrogenase from *Plasmodium falciparum*
**1**	7.1	7.5
**2**	6.8	8.9
Benznidazole (standard) ^a^	5.6	-
Chloroquine (standard) ^b^	-	6.3

(Standard) ^a^ for antitrypanosomal activity. (Standard) ^b^ for antiplasmodium activity.

**Table 3:** In vitro antitrypanosomal and antimalarial activities of **1** and **2**.

Compounds	IC_50_ (μM)	
	Antitrypanosomal Activity *Trypanosoma cruzi* C2C4 Strain	Antimalarial Activity *Plasmodium falciparum* 3D7 Strain
**1**	9.6	2.3
**2**	>50	>50
Benznidazole ^[a]^	2.6	NA
Chloroquine ^[b]^	NA	0.017

^[a]^ Benznidazole is the standard for the *T. cruzi* C2C4 strain. ^[b]^ Chloroquine is the standard for the *P. falciparum* 3D7 strain. NA, not applicable.

## Data Availability

Not applicable.

## Supplementary Materials

The following supporting information can be downloaded at: https://www.mdpi.com/article/10.3390/antibiotics12010109/s1, Figure S1: Structure of fumitremorgin C (**1**); Figure S2: ESI MS (positive ionisation mode) of fumitremorgin C (**1**); Figure S3: 1H NMR spectrum of fumitremorgin C (**1**) at 600 MHz in CD3OD; Figure S4: HSQC spectrum of fumitremorgin C (**1**) at 600 MHz in CD3OD; Figure S5: COSY spectrum of fumitremorgin C (**1**) at 600 MHz in CD3OD; Figure S6: HMBC spectrum of fumitremorgin C (**1**) at 600 MHz in CD3OD; Figure S7: Structure of pseurotin D (**2**); Figure S8: ESI MS (positive ionisation mode) of pseurotin D (**2**); Figure S9: 1H NMR spectrum of pseurotin D (**2**) at 600 MHz in CD3OD; Figure S10: HSQC spectrum of pseurotin D (**2**) at 600 MHz in CD3OD; Figure S11: COSY spectrum of pseurotin D (**2**) at 600 MHz in CD3OD; Figure S12: HMBC spectrum of pseurotin D (**2**) at 600 MHz in CD3OD; Table S1: NMR data for fumitremorgin C (**1**); Table S2: NMR data for pseurotin D (**2**).
