# Supplementary material for: Antiparasitic Activities of Compounds Isolated from Aspergillus fumigatus Strain Discovered in Northcentral Nigeria
Source: Antibiotics (Basel). 2023 Jan 6;12(1):109. doi: 10.3390/antibiotics12010109 (PMC9854968; doi:10.3390/antibiotics12010109)
Supplement: Supplementary file 1 [file antibiotics-12-00109-s001.zip › antibiotics-2086856-supplementary.pdf]

Table S1. NMR data for fumitremorgin C (**1**) [1], [2]

**Position**	**δC, mult.**	**δC (Reported)**	**δH, mult. (*J* in Hz)**	**COSY**	**HMBC (H→C)**
1-NH					
2	133.5, C	133.6			
3	51.9, CH	52.4	6.0, d (*J* = 9.6 Hz)	21	5, 2, 22, 21, 14, 12
4- N					
5	171.5, C	171.6			
6	60.2, CH	60.4	4.27, dd (*J* = 12.6, 6.5 Hz)	7	**11**
7, 7′	29.0, CH_2	29.5	2.4, 2.1, m	6, 8	5, 9
8	23.5, CH_2	24.0	2.1, m	7, 9	6
9	46.1, CH_2	46.4	3.6, m	8	
10-N					
11	167.8, C	168.1			
12	57.7, CH	57.9	4.30, dd (*J* = 12.6, 6.5 Hz)	13	
13, 13′	22.2, CH_2	22.8	3.58, m ; 3.0, dd (*J* = 15.7, 11.6 Hz)	12	2, 11, 12, 14, 15
14	106.0, C	106.1			
15	121.0, C	121.9			
16	119.1, CH	119.2	7.4, d (J = 6.8 Hz)	17	15, 14,18, 20
17	109.7, CH	110.1	6.77, dd (*J* = 8.6, 2.3 Hz)	16, 19	15, 19
18	157.5, C	157.6			
19	94.2, CH	95.9	6.87, d (*J* = 2.3 Hz)	17	15, 17, 18, 20
20	138.8, C	138.9			
21	125.1, CH	125.2	4.9, d (*J* = 9.6 Hz)	3	w
22	135.1, C	135.1			
23	25.3, CH_3	25.9	1.7, s		2, 21, 24
24	18.0, CH_3	18.3	2.0, s		2, 21, 23
25	55.8, OCH_3	55.9	3.8, s		18

NMR solvents used for fumitremorgin C (**1**) and that reported in the literature were CD_3OD and $CDCl_3$ at 600 MHz and 500 MHz, respectively. w= weak HMBC intensity.

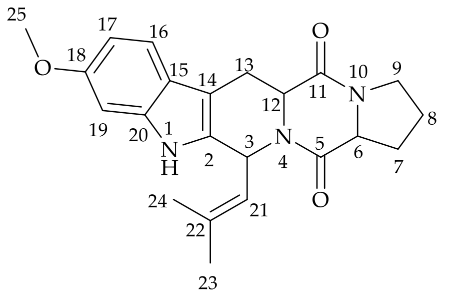

Chemical Formula: $C_{22}H_{25}N_3O_3$
Exact Mass: 379.1896

Figure S1. Structure of fumitremorgin C (**1**)

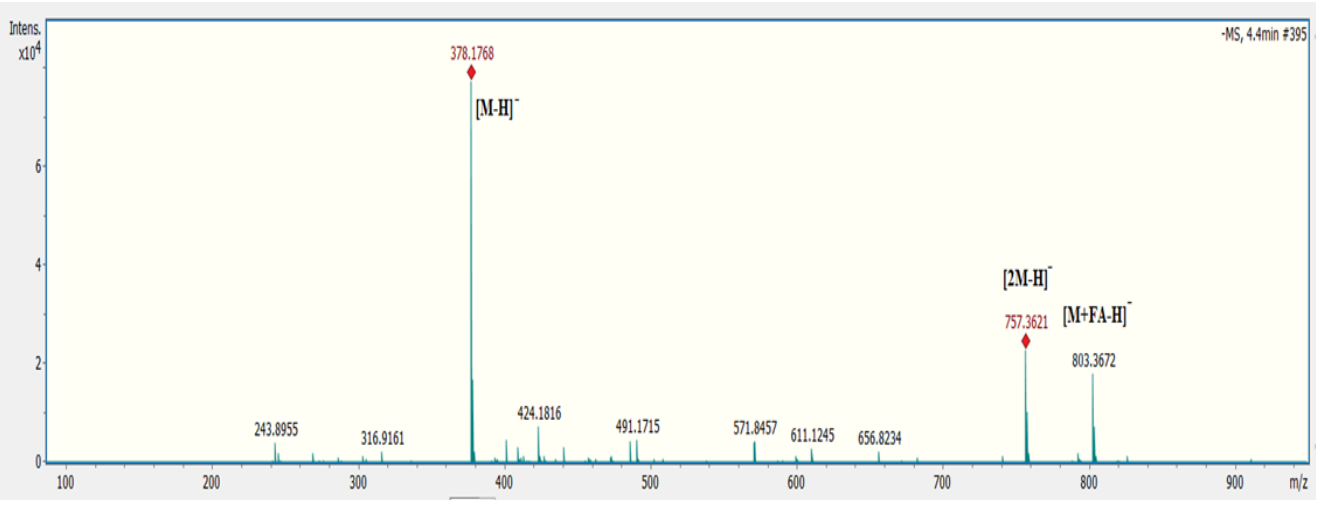

Figure S2. ESI MS (positive ionisation mode) of fumitremorgin C (**1**)

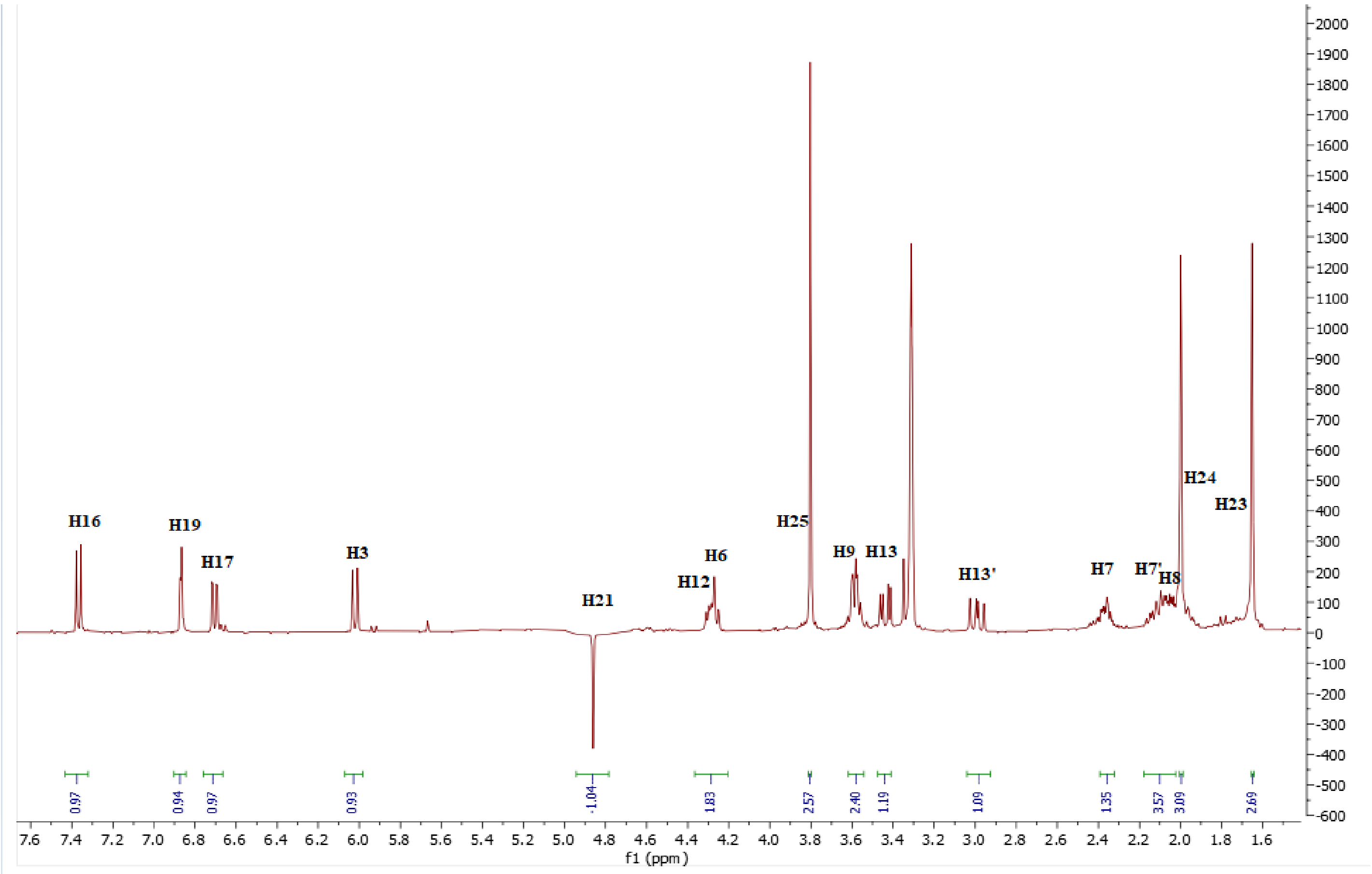

Figure S3. ^{1}H NMR spectrum of fumitremorgin C (**1**) at 600 MHz in CD_3OD

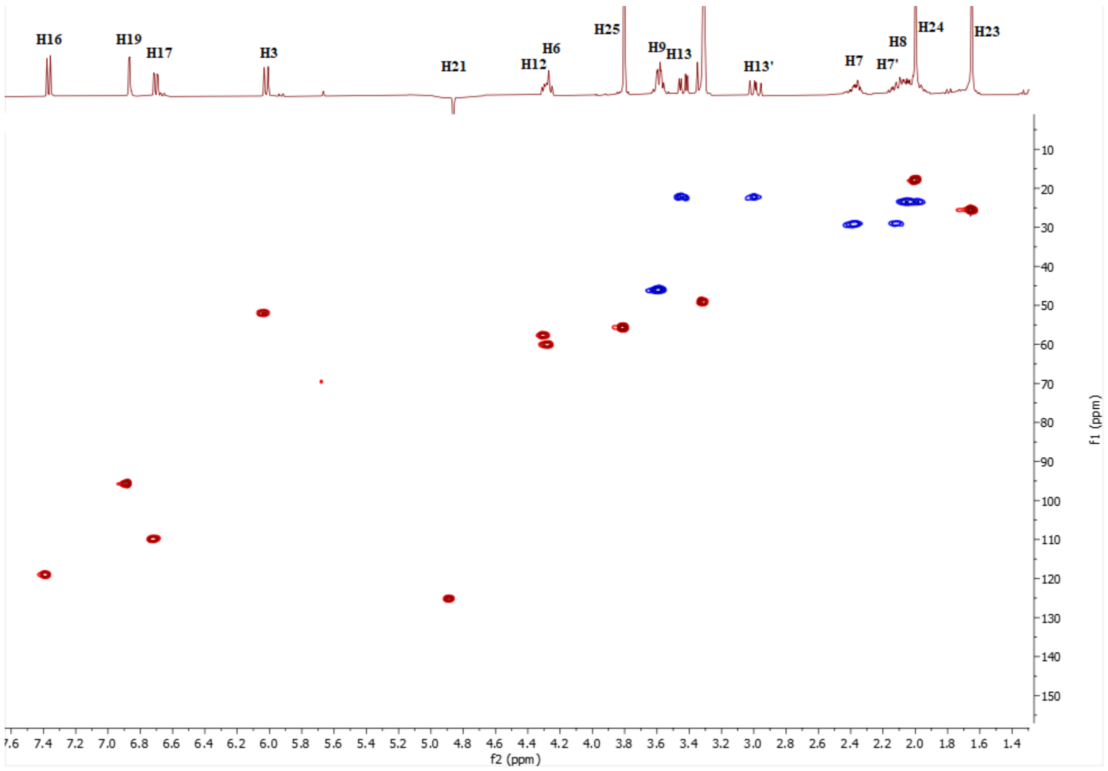

Figure S4. HSQC spectrum of fumitremorgin C (**1**) at 600 MHz in CD_3OD

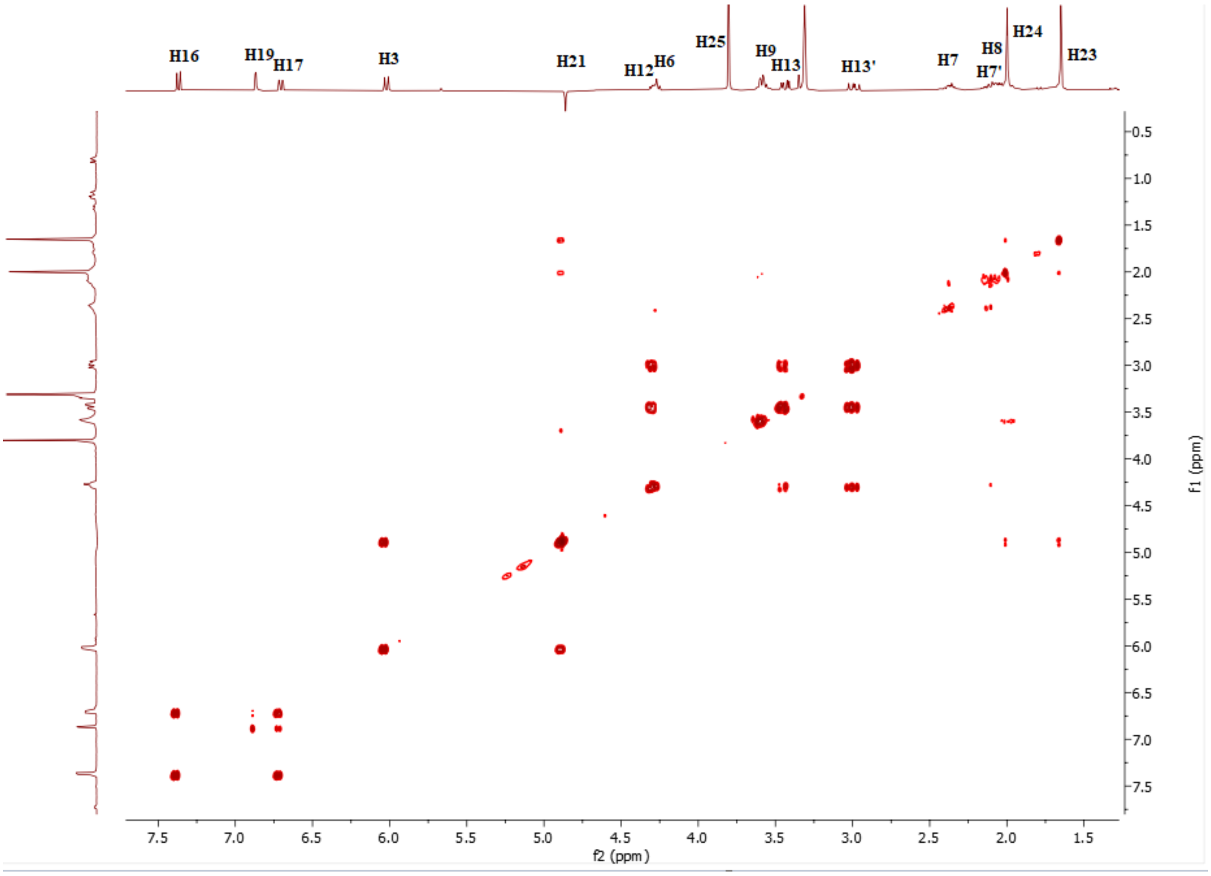

Figure S5. COSY spectrum of fumitremorgin C (**1**) at 600 MHz in CD_3OD

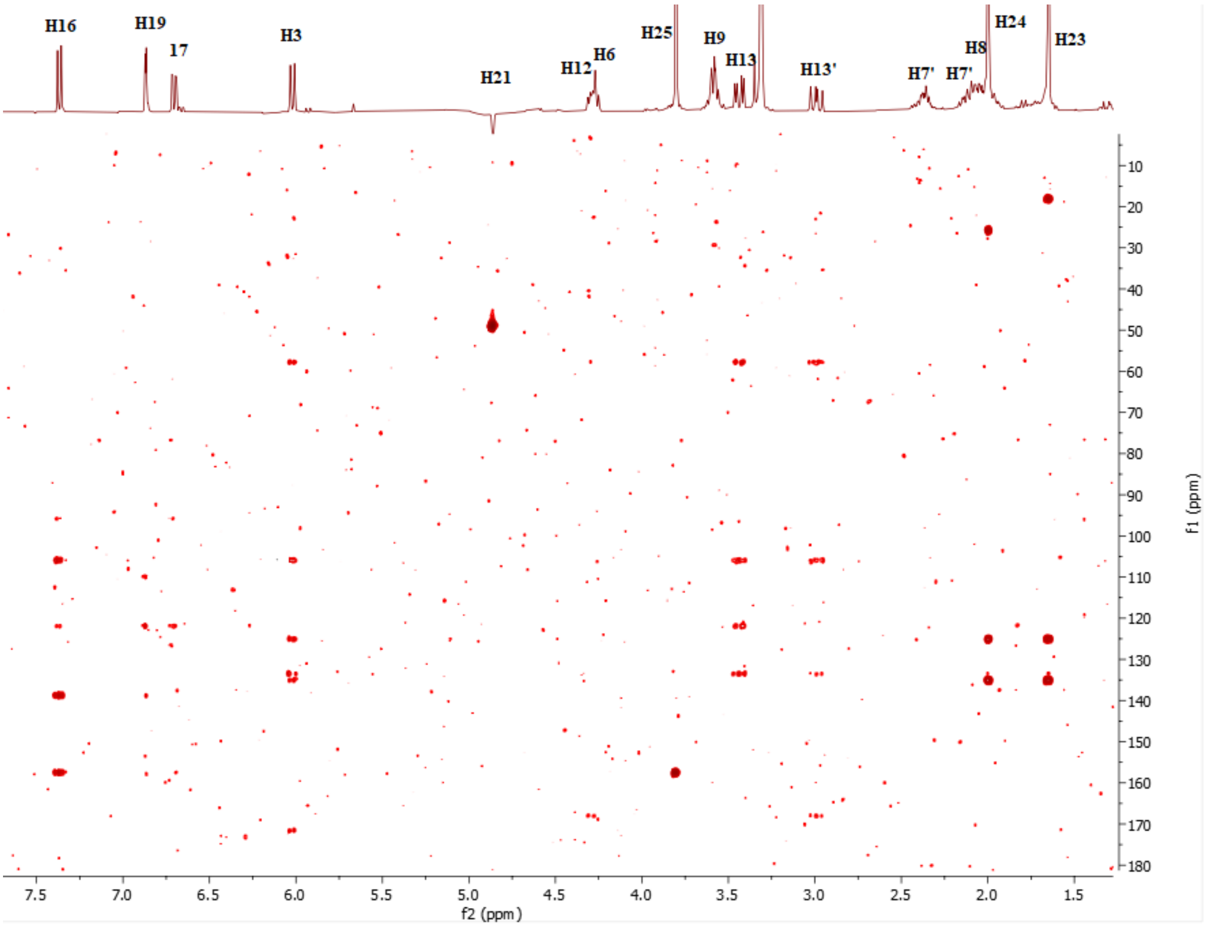

Figure S6. HMBC spectrum of fumitremorgin C (**1**) at 600 MHz in CD_3OD

Table S2. NMR data for pseurotin D (**2**) [3]

**Position**	**δC, mult.**	**δC reported**	**δH, mult. (*J* in Hz)**	**HMBC (H→C)**
2	189.1, C	187.8		
3	112.1, C	111.2		
4	199.3, C	197.4		
5	93.6, C	91.0		
6	168.8, C	166.8		
8	93.8, C	91.9		
9	76.3, CH	74.1	4.52, d (*J* = 2.3 Hz)	4, 8
10	69.8, CH	68.7	5.20, dd (*J* = 6.1, 3.5 Hz)	2, 3, 11, 12
11	127.9, CH	126.4	5.87, m	2, 10, 11, 13
12	137.9, CH	137.3	5.94, m	10, 12, 13, 14
13	74.0, CH	73.1	4.02, q (*J* = 6.3 Hz)	11, 12, 14, 15
14	30.9, CH	29.7	1.52, m	12, 13, 16
15	5.5, CH_3	5.4	0.91, s	
16	10.1, CH_3	9.8	1.76, s	
17	52.5, OCH_3	52.0	3.36, s	8
18	197.1, C	195.2		
19	139.2, C	134.6		
20, 24	131.7, CH	130.8	8.37, d (*J* = 7.4 Hz)	18, 22
21, 23	129.5, CH	128.7	7.52, t (*J* = 7.4 Hz)	19
22	134.8, CH	132.7	7.66, t (*J* = 7.4 Hz)	20, 24

NMR solvents used for pseurotin D (**2**) and that reported in the literature were CD_3OD and $CDCl_3$ at 600 MHz, respectively.

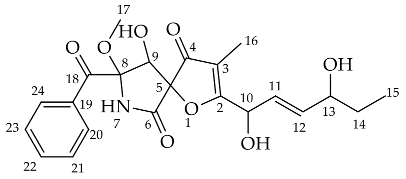

Chemical Formula: $C_{22}H_{25}NO_8$
Exact Mass: 431.1580

Figure S7. Structure of pseurotin D (**2**)

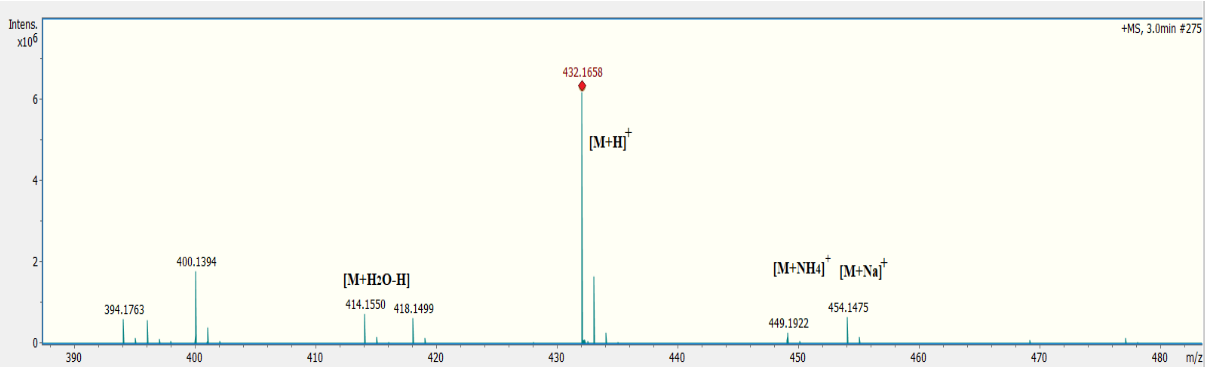

Figure S8. ESI MS (positive ionisation mode) of pseurotin D (**2**)

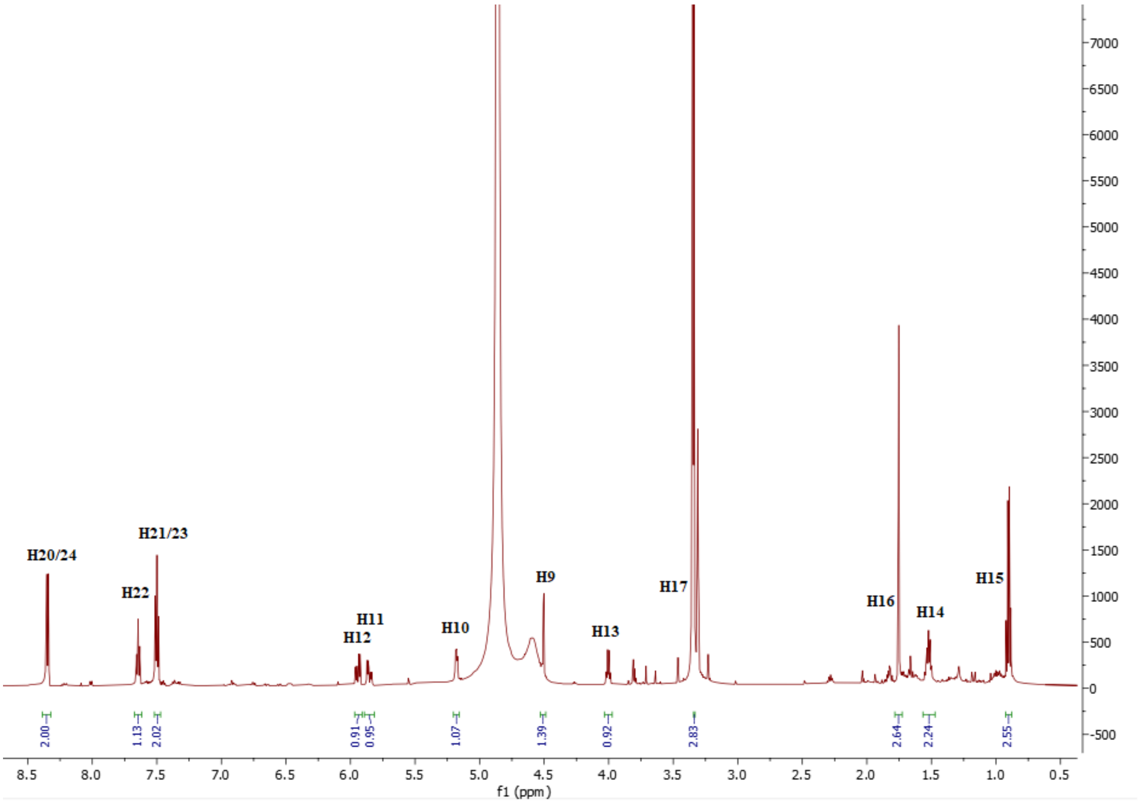

Figure S9. ^{1}H NMR spectrum of pseurotin D (**2**) at 600 MHz in CD_3OD

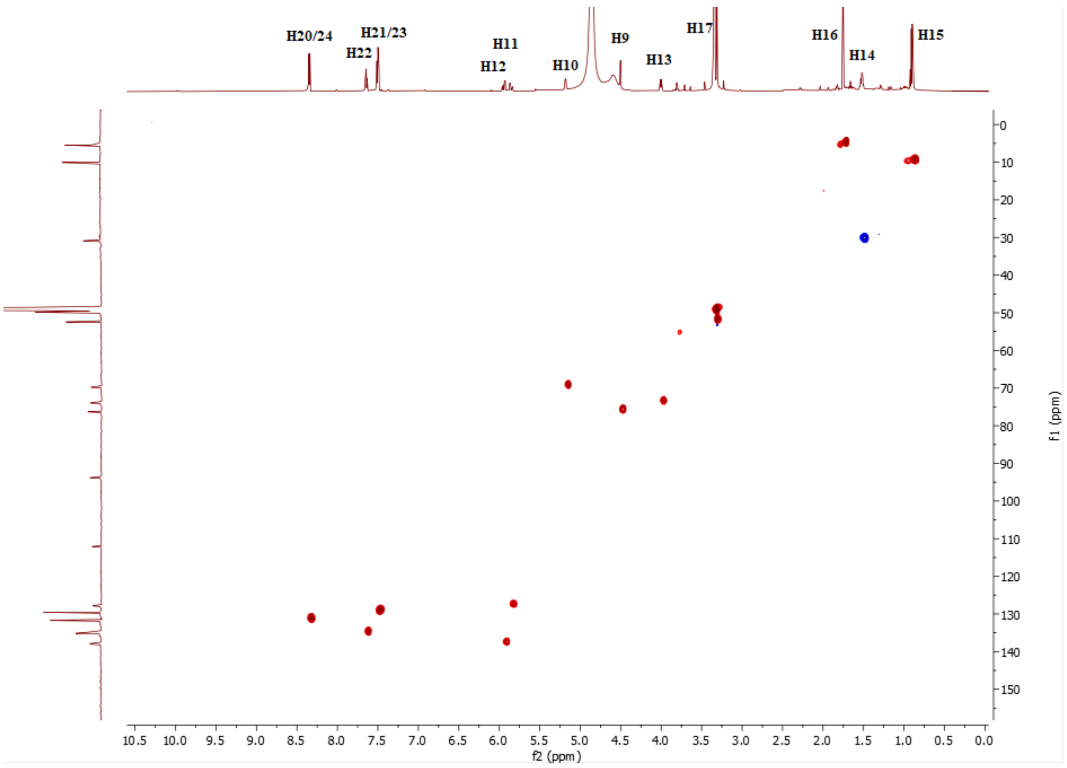

Figure S10. HSQC spectrum of pseurotin D (**2**) at 600 MHz in CD_3OD

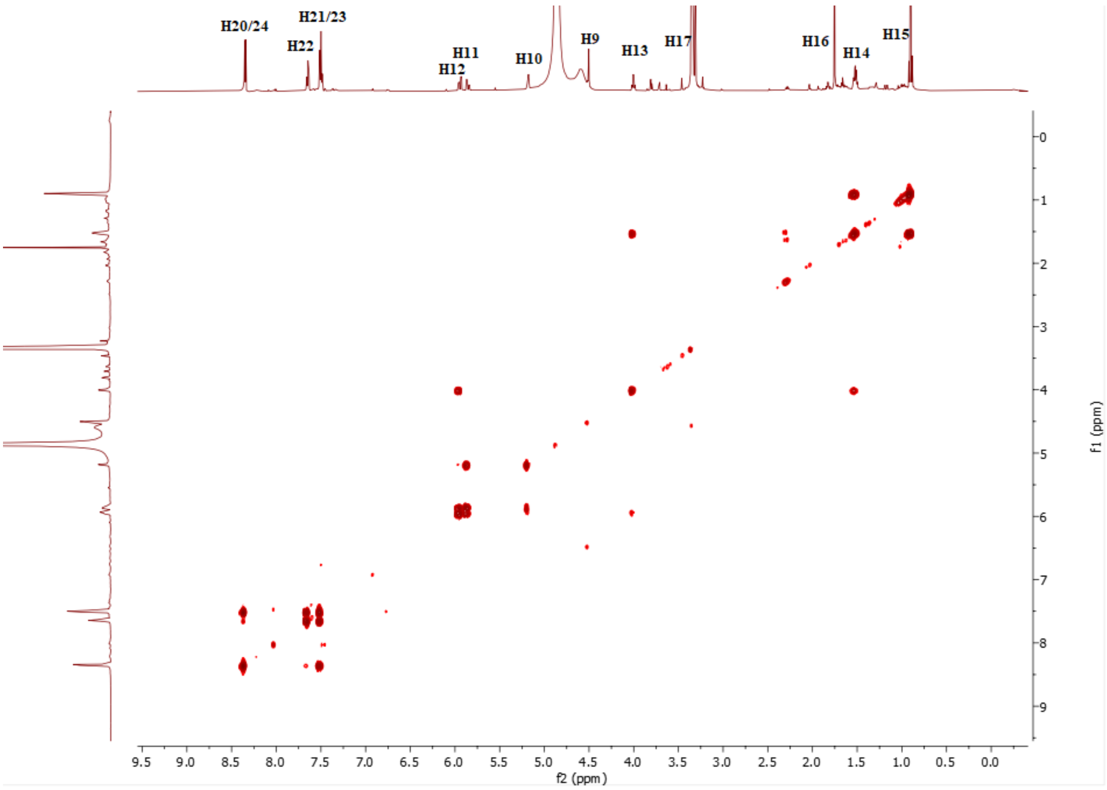

Figure S11. COSY spectrum of pseurotin D (**2**) at 600 MHz in CD_3OD

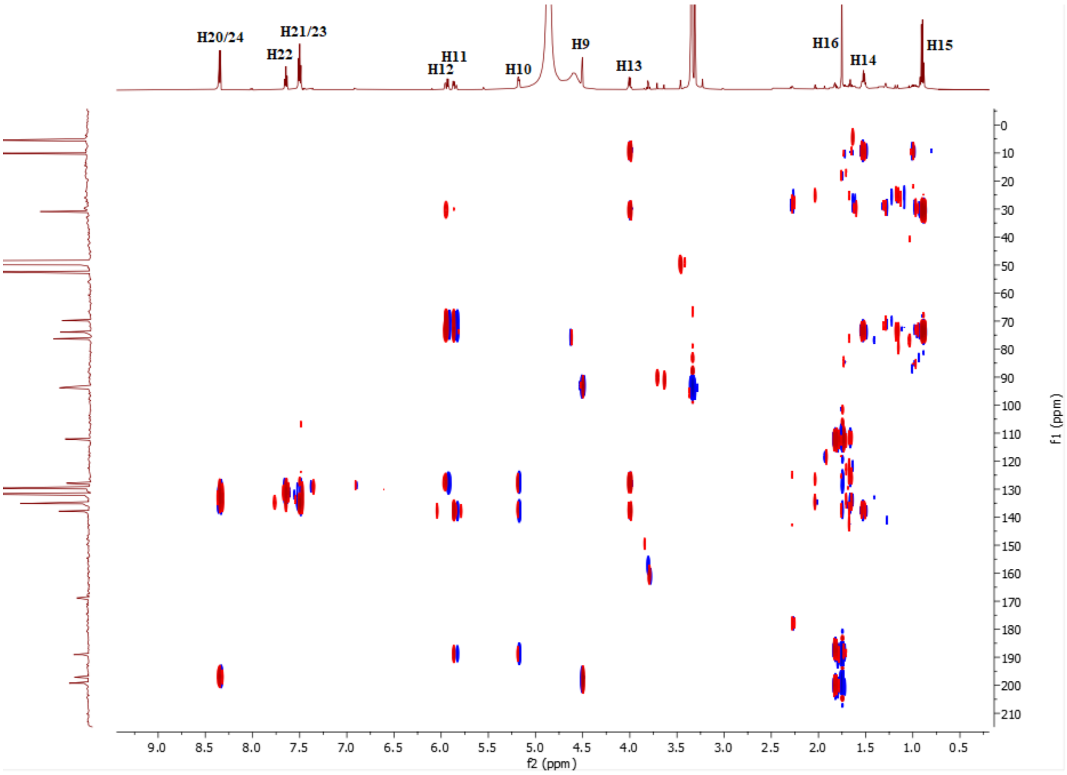

Figure S12. HMBC spectrum of pseurotin D (**2**) at 600 MHz in CD_3OD

References

[1] X. Li *et al*, "Metabolites from *Aspergillus fumigatus*, an endophytic fungus associated with *Melia azedarach*, and their antifungal, antifeedant, and toxic activities," *J. Agric. Food Chem.*, vol. 60, *(13)*, pp. 3424-3431, 2012.

[2] Q. Lu *et al*, "Bioactive metabolites from the mycelia of the basidiomycete *Hericium erinaceum*," *Natural Product Research*, vol. 28, *(16)*, pp. 1288-1292, 2014.

[3] D. Copmans *et al*, "Zebrafish-based discovery of antiseizure compounds from the Red Sea: pseurotin A2 and azaspirofuran A," *ACS Chemical Neuroscience*, vol. 9, *(7)*, pp. 1652-1662, 2018.
